# PARP inhibitors affect growth, survival and radiation susceptibility of human alveolar and embryonal rhabdomyosarcoma cell lines

**DOI:** 10.1007/s00432-018-2774-6

**Published:** 2018-10-24

**Authors:** Simona Camero, Simona Ceccarelli, Francesca De Felice, Francesco Marampon, Olga Mannarino, Lucrezia Camicia, Enrica Vescarelli, Paola Pontecorvi, Barry Pizer, Rajeev Shukla, Amalia Schiavetti, Maria Giovanna Mollace, Antonio Pizzuti, Vincenzo Tombolini, Cinzia Marchese, Francesca Megiorni, Carlo Dominici

**Affiliations:** 1 Department of Paediatrics, “Sapienza” University of Rome, Viale Regina Elena 324, 00161 Rome, Italy; 2 Department of Experimental Medicine, “Sapienza” University of Rome, Rome, Italy; 3 Department of Radiological, Oncological and Pathological Sciences, “Sapienza” University of Rome, Rome, Italy; 4 Department of Oncology, Alder Hey Children’s NHS Foundation Trust, Eaton Road, Liverpool, L12 2AP UK; 5 Department of Perinatal and Paediatric Pathology, Alder Hey Children’s NHS Foundation Trust, Liverpool, UK

**Keywords:** Rhabdomyosarcoma, PARP inhibitors, Olaparib, AZD2461, Radiosensitivity

## Abstract

**Purpose:**

PARP inhibitors (PARPi) are used in a wide range of human solid tumours but a limited evidence is reported in rhabdomyosarcoma (RMS), the most frequent childhood soft-tissue sarcoma. The cellular and molecular effects of Olaparib, a specific PARP1/2 inhibitor, and AZD2461, a newly synthesized PARP1/2/3 inhibitor, were assessed in alveolar and embryonal RMS cells both as single-agent and in combination with ionizing radiation (IR).

**Methods:**

Cell viability was monitored by trypan blue exclusion dye assays. Cell cycle progression and apoptosis were measured by flow cytometry, and alterations of specific molecular markers were investigated by, Real Time PCR, Western blotting and immunofluorescence experiments. Irradiations were carried out at a dose rate of 2 Gy (190 UM/min) or 4 Gy (380 UM/min). Radiosensitivity was assessed by using clonogenic assays.

**Results:**

Olaparib and AZD2461 dose-dependently reduced growth of both RH30 and RD cells by arresting growth at G2/M phase and by modulating the expression, activation and subcellular localization of specific cell cycle regulators. Downregulation of phospho-AKT levels and accumulation of γH2AX, a specific marker of DNA damage, were significantly and persistently induced by Olaparib and AZD2461 exposure, this leading to apoptosis-related cell death. Both PARPi significantly enhanced the effects of IR by accumulating DNA damage, increasing G2 arrest and drastically reducing the clonogenic capacity of RMS-cotreated cells.

**Conclusions:**

This study suggests that the combined exposure to PARPi and IR might display a role in the treatment of RMS tumours compared with single-agent exposure, since stronger cytotoxic effects are induced, and compensatory survival mechanisms are prevented.

**Electronic supplementary material:**

The online version of this article (10.1007/s00432-018-2774-6) contains supplementary material, which is available to authorized users.

## Introduction

Rhabdomyosarcoma (RMS) is the most common childhood soft tissue sarcoma, representing approximately 50% of all sarcomas in children aged 0–14 years (McDowell 2003; O’Neill et al. 2013). Adolescents and more rarely adults may also be affected (Ferrari et al. 2003). RMS is a heterogeneous tumour that is believed to develop as a result of genetic alterations occurring in mesenchymal progenitor/stem cells, which express some markers of normal skeletal muscle but show an incompletely differentiated muscle phenotype (Merlino and Helman 1999). Alveolar RMS (ARMS) and embryonal RMS (ERMS), the two most common histological subtypes in childhood, have distinct clinicopathological features and outcomes (Coffin 1997; Parham and Barr 2013). ARMSs and ERMSs are both characterised by distinctive genetic alterations that are likely to play a decisive role in their pathogenesis (Anderson et al. 1999; Goldstein et al. 2006; Martinelli et al. 2009; Marshall and Grosveld 2012; Parham and Barr 2013; Robbins et al. 2016). ERMSs are more frequent (~ 80% of cases) and generally affect younger children (0–4 years), occurring more commonly in the neck, head and genito-urinary tract (Parham and Barr 2013). ARMSs (~ 20% of cases) usually present throughout childhood and adolescence, frequently originate in the extremities and trunk, often with regional or metastatic lymph node involvement already at diagnosis, and have high tendency to metastasize carrying a significantly worse outcome (Parham and Barr 2013). Indeed, 70% of children with localized disease survive with conventional treatment (Arndt et al. 2009), including surgery, radiotherapy and chemotherapy. Metastatic RMSs, however, are frequently resistant or present relapse after an initial response, with a 5-year event-free survival rate at about 30% (Sorensen et al. 2002; Ognjanovic et al. 2009; Wolden et al. 2015). Therefore, the outcome for high-risk RMS cases remains very poor and the discovery of innovative therapies is an absolute priority to improve therapeutic activity and reduce toxicity.

Poly(ADP-ribose) polymerases (PARPs) belong to a large family of enzymes that catalyse the formation of poly(ADP-ribose) polymers (PARylation) onto different targets and themselves, this leading to a fine modulation of different cellular processes and molecular pathways, such as DNA damage response (DDR), cellular differentiation, chromatin remodelling, transcription, cell death and mitotic progression (Helleday et al. 2007; Dungey et al. 2008; De Vos et al. 2012; Bai 2015; Brown et al. 2017). Only PARP1 and, to a lesser extent, PARP2 and PARP3 play an essential role in repairing single- or double-stranded DNA breaks (SSBs or DSBs, respectively) as well as stalled replication forks and DNA crosslinks (De Vos et al. 2012), with PARP2 being specifically able to recognize DNA gaps and flaps (Yélamos et al. 2008) and PARP3 being selectively activated by DSBs (Boehler et al. 2011). PARP1 and PARP2 are involved in fixing DNA-strand interruptions by the homologous recombination (HR) pathway (Henning and Stürzbecher 2003), whilst PARP3 acts via the nonhomologous end joining (NHEJ) repair system (Davis and Chen 2013).

PARP inhibitors (PARPi) comprehend a wide range of chemical compounds able to abrogate PARP functionality thus bringing to the accumulation of SSBs, which in turn are converted into DSBs that cells are not able to repair causing cancer cell death (Wiltshire et al. 2010). The mechanism of action of PARPi is the block of the catalytic domain of PARP enzymes, but these agents can also trap PARP proteins on the double-stranded DNA helix, this leading to cytotoxic lesions (Murai et al. 2012; D’Arcangelo et al. 2016). PARP inhibition has a potential therapeutic role as monotherapy in tumours carrying constitutive mutations in DDR genes, as well as in combination therapies for its ability to enhance the activity of anticancer drugs with genotoxic action, including DNA alkylating agents, topoisomerase II inhibitors and ionising radiation (IR) (Jorgensen 2009; Kelley et al. 2014; Lord and Ashworth 2017), since targeting similar molecular functions results in cell death. The “synthetic lethality” conferred by PARPi (Martin et al. 2008; Lord and Ashworth 2017) is not only restricted to BRCA1- and BRCA2-mutated tumours but also to neoplasia harbouring genetic alterations in other HR genes, such as ATM, RAD51, PTEN, XRCC2, etc (Bang et al. 2013; Kelley et al. 2014), this suggesting a therapeutic potential role of PARP inhibition in a wide range of human malignancies. Several clinical trials aimed at assessing for different PARPi are in progress. Olaparib (AZD2281), a selective inhibitor of PARP1 and PARP2, has been used in different solid tumours and, recently, this drug has been approved as personalized therapy (Kim et al. 2015; Goulooze et al. 2016) for patients with BRCA1/2-mutated advanced ovarian cancer, who have been treated with three or more prior lines of chemotherapy (Phase III clinical trial, NCT01874353). AZD2461, a next-generation agent able to also inhibit PARP3 activity, has been recently synthesised in order to overcome PARPi-related resistance and to be better tolerated than Olaparib (Jaspers et al. 2013; O’Connor et al. 2016; Vaidyanathan et al. 2016). An encouraging therapeutic activity has recently been reported in clinical trials with AZD2461 on refractory solid tumours (Phase I clinical trial, NCT01247168).

A limited amount of information is available about the effects and the molecular mechanisms of PARP inhibition, as monotherapy or in combination with conventional therapies, in RMS. Only very recently, Mangoni et al. have shown that pretreatment with Olaparib, Iniparib or Veliparib, three PARP1 inhibitors, is able to induce a significant radiosensitization in different soft tissue sarcoma cell lines (Mangoni et al. 2018).

In the present study, we analysed the expression of PARP1, PARP2 and PARP3 genes in a panel of RMS primary tumours and cell lines, and evaluated the biological and molecular effects of PARP inhibition in RMS in vitro models by using Olaparib or AZD2461. We tested two different doses of both PARPi and determined the minimum concentrations of each molecule able to drive a biological effect in RH30 and RD cell lines, two in vitro models of ARMS and ERMS, respectively. We also assessed the possible synergistic effects between Olaparib or AZD2461 and IR, a combination which might represent a further step towards a more effective treatment of RMS patients, especially those with metastatic disease.

## Methods

### Reagents and irradiation

Olaparib and AZD2461 were purchased from Selleckchem (Suffolk, UK) and were reconstituted at 10 mM using dimethyl sulfoxide (DMSO). DMSO alone was used as control in untreated cells at 0.1% (v/v) concentration.

Irradiation was carried out using an ONCOR Impression Linear Accelerator (Siemens Medical Solutions USA, Inc, Concord, CA) at a dose rate of 2 Gy (190 UM/min) or 4 Gy (380 UM/min).

### Cell cultures

Human RMS cell lines, RH30 (alveolar) and RD (embryonal), were maintained as previously described (Megiorni et al. 2016). Human foetal myoblast (HFM) cells were cultured in High Glucose DMEM supplemented with 20% FBS.

### Tumour samples

Seventeen RMS tumour samples, 4 ARMSs and 13 ERMSs, were obtained at diagnosis before any treatment from children admitted to the Department of Oncology at Alder Hey Children’s NHS Trust, Liverpool. ARMS1-2-4 are fusion-positive tumours, whilst ARMS3 is a fusion-negative case, as assessed by FISH analysis for PAX3/7-FOXO1 translocations. Institutional written informed consent was obtained from the patient’s parents or legal guardians. The study underwent ethical review and approval according to the local institutional guidelines (Alder Hey Children’s NHS Foundation Trust Ethics Committee, approval number 09/H1002/88).

### RNA extraction and quantitative Real Time PCR (q-PCR)

Total RNA, isolated from RMS tumour biopsies and cell lines, was reverse transcribed and analysed by using quantitative Real Time PCR (q-PCR) with specific TaqMan Real-Time Gene Expression Assays (Applied Biosystems), as previously described (Megiorni et al. 2016). Human PARP1 (Hs00242302_m1), PARP2 (Hs00193931_m1) and PARP3 (Hs00193946_m1) mRNA assays were used. Samples were normalized according to GAPDH transcript levels. Expression of miR-124-3p was analysed as previously described (Megiorni et al. 2014), by using sequence-specific TaqMan MicroRNA Assays (Applied Biosystems). U6 small nuclear RNA levels were used as internal control. The amount of each mRNA or miRNA was calculated by the comparative *C*_t_ method (Livak and Schmittgen 2001) and expressed as fold change using the StepOne v2.3 software (Applied Biosystems). Each sample was run in triplicate, in at least two independent experiments.

### Cell proliferation assays

RH30 and RD cells (3 × 10^5^) were plated in six-well plates and treated with Olaparib (1.5 and 5 µM) or AZD2461 (5 and 10 µM). After 72 h, RH30 and RD living cells were diluted in a 1:1 mixture of trypan blue (Invitrogen) and counted using the Countess II Automated Cell Counter (Invitrogen), according to the manufacturer’s instructions.

### Morphological assessment

RH30 and RD cells treated with Olaparib (1.5 and 5 µM) or AZD2461 (5 and 10 µM) for 72 h were photographed with an Axio Vert.A1 microscope (Carl Zeiss Microscopy, Thornwood, NY), furnished with an AxioCam MRc5 camera (Carl Zeiss Microscopy), at 20× magnification.

### Cell cycle and apoptosis analysis by flow cytometry

For the cell cycle analysis, RH30 and RD cells (3 × 10^5^) were incubated in six-well cell culture plates overnight to allow cell adhesion. Cells were treated with Olaparib (1.5 and 5 µM) or AZD2461 (5 and 10 µM) for 48 h. DMSO was used as mocked control. For the cell cycle analysis of the effects induced by the PARPi and IR combination, RH30 and RD cells, pretreated for 24 h with Olaparib or AZD2461 were irradiated and incubated for additional 24 h at 37 °C. Samples were stained with Propidium Iodide (PI) solution and subjected to flow cytometry by using a BD FACSCalibur (BD Biosciences, Franklin Lakes, NJ), as previously described (Megiorni et al. 2016). FACS data were quantified by using the ModFit LT 3.0 program (Verity Software House). Experiments were performed at least twice.

Apoptosis was analysed by using PE Annexin V Apoptosis Detection Kit I (BD Biosciences), following the manufacturer’s instructions. Briefly, RH30 and RD cells (3 × 10^5^) were seeded overnight in six-well plate and treated with Olaparib, AZD2461 or DMSO for 48 and 144 h. Approximately 2 × 10^5^ cells were stained with Annexin V and 7-Amino-Actinomycin (7-AAD) for 15 min at RT in the dark. Fluorescence intensities of treated samples and controls were analysed by flow cytometry by using the BD CellQuest Pro software. Experiments were performed at least twice.

### Colony formation assay

RH30 and RD cells (3.2 × 10^5^) treated for 24 h with Olaparib (1.5 and 5 µM) or AZD2461 (5 and 10 µM), were irradiated at a dose of 2 or 4 Gy/min. After 4 h incubation at 37 °C, 5% CO_2_, 2 × 10^3^ cells/well were seeded in 6-well plates in triplicate. Medium was replaced every 3 days and after 12 days, colonies were stained with 0.1% crystal violet for 5 min at room temperature (RT). Colonies were photographed, and then crystal violet was solubilised in 30% acetic acid in water for 15 min at RT; absorbance was measured by using the Biochrom Libra S22 UV/VIS spectrophotometer (Biochrom, Berlin, DE) at wavelength of 595 nm; 30% acetic acid in water was used as the blank. Colony formation capacity in PARPi- and/or IR-treated cells was calculated in comparison to mocked control samples (DMSO alone), arbitrarily set to 1. The results were plotted as means ± SD of two separate experiments having three determinations per assay for each experimental condition.

### Protein extraction and Western blotting

Total protein extracts and Western blotting assays were performed as previously described (Megiorni et al. 2016) using the following primary antibodies: phospho (p)-AKT, AKT, cleaved caspase 3, and γH2AX (Cell Signalling Technology, Danvers, MA); Bcl2, Cdc2 phosphorylated at Thr14/Tyr15, Cdc25C, Cyclin B1, Cyclin D1 and p21 (Santa Cruz Biotechnology, Dallas, TX). Antibody against tubulin (Sigma-Aldrich) was used as a loading control.

### Immunofluorescence (IF) microscopy

RH30 and RD cells (5 × 10^4^), seeded onto 2% gelatine coated-glass coverslips in 24-well plates, were allowed to attach overnight and then incubated for 48 h in the presence or absence of Olaparib (5 µM) or AZD2461 (10 µM). For IF analysis of the effects induced by the PARPi and IR combination, RH30 and RD cells, pretreated for 24 h with Olaparib or AZD2461 were irradiated and incubated for additional 4 h at 37 °C. IF assays were performed as previously described (Megiorni et al. 2016) using the following primary antibodies: Cdc2, p-Cdc2, Cdc25C, Cyclin B1, Cyclin D1, RAD51 (1:20 dilution in PBS; Santa Cruz Biotechnology), and γH2AX (1:500 in PBS; Cell Signaling). All single-stained or merged images were acquired with a Zeiss ApoTome microscope (40× magnification) using the Axiovision software (Carl Zeiss, Jena, Germany). For γH2AX and RAD51, focus fluorescence intensity in the respect of cell number in each analysed field was reported.

### Statistical analysis

Data are presented as means ± SD. Statistical analyses were performed by two-tailed Student’s *t* test and a probability (*p*) < 0.05 was considered statistically significant. All the experiments were done in triplicates and repeated three times unless mentioned otherwise.

## Results

### PARP1, PARP2 and PARP3 are upregulated in ARMS and ERMS tumours and cell lines

**Fig. 1:**
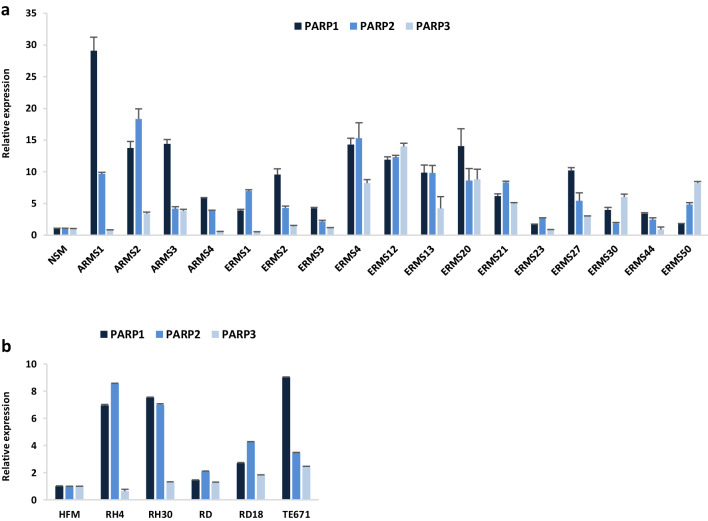
PARP1, PARP2 and PARP3 expression in RMS tumours and cell lines. **a** Quantitative real time PCR (q-PCR) analysis of PARP1, PARP2 and PARP3 mRNA levels in 17 RMS primary tumours (4 ARMSs and 13 ERMSs), expressed as fold increase over normal skeletal muscle (NSM), arbitrarily set at 1. Transcript levels were normalized to GAPDH mRNA and error bars represent SD of two independent q-PCR reactions, each performed in triplicate. **b** q-PCR of PARP1, PARP2 and PARP3 mRNA levels in ARMS (RH4 and RH30) and ERMS (RD, RD18 and TE671) cell lines, expressed as fold increase over HFM (human foetal myoblast), arbitrarily set at 1. GAPDH was used as control. Bars represent mean values of two independent experiments, each performed in triplicate

Transcript levels of PARP1, PARP2 and PARP3 genes were evaluated in a panel of 17 RMS primary tumours (4 ARMSs and 13 ERMSs) by quantitative Real Time PCR (q-PCR) experiments. PARP1, PARP2 and PARP3 (Fig. 1a) mRNAs are all significantly overexpressed in RMS biopsies compared with normal skeletal muscle (NSM), with a 9.3-, 7.1- and 4.2-fold average increase in PARP1, PARP2 and PARP3 expression, respectively.

PARP expression was also analysed in two ARMS (RH4 and RH30) and three ERMS (RD, RD18 and TE671) cell lines, and higher levels of PARP1, PARP2 and PARP3 transcripts were demonstrated in comparison to proliferating myoblasts (HFMs), with the only exception of PARP3 in RH4 cell line (Fig. 1b). Aberrant expression of PARPs in RMS suggests that these genes might be further investigated as potential therapeutic targets in RMS tumours.

### Olaparib and AZD2461 inhibit cell proliferation in RMS cell lines by inducing cell cycle arrest in G2/M phase

**Fig. 2:**
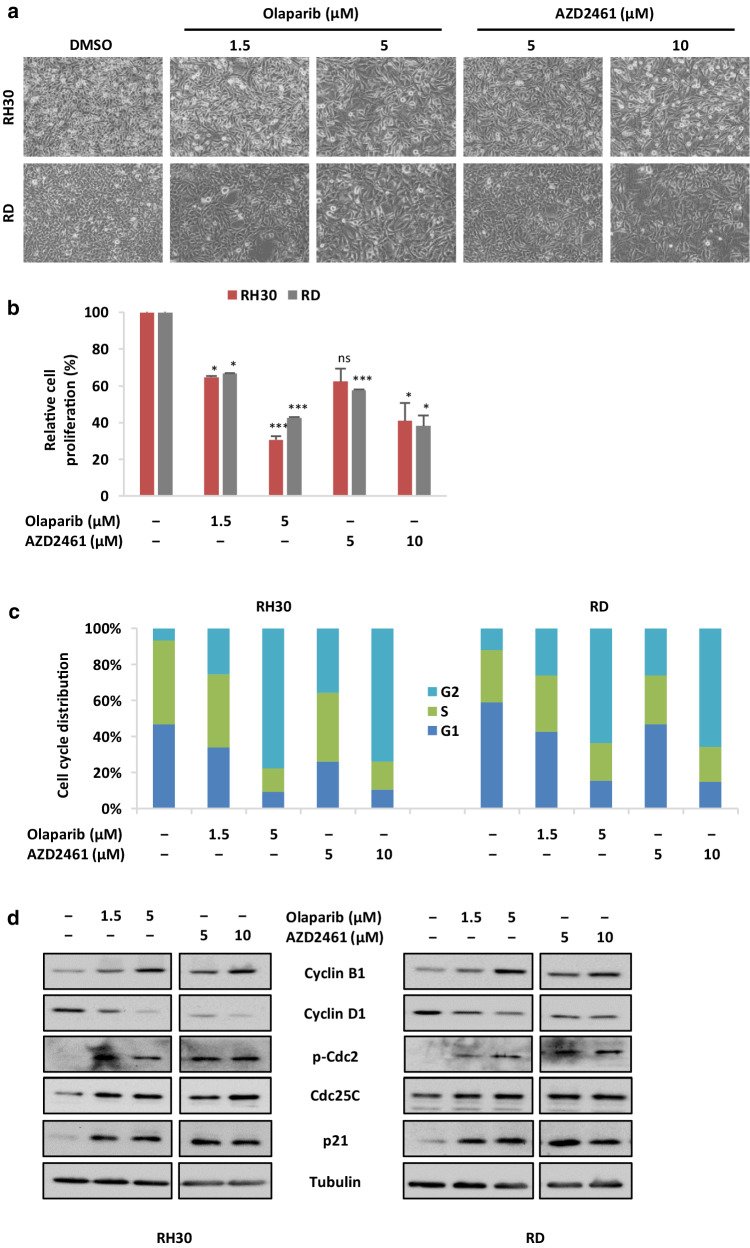
PARPi exposure affects cell viability and induces G2/M cell cycle arrest in RMS cell lines. **a** Olaparib or AZD2461 treatment for 48 h clearly affected the morphology of both RH30 and RD cells, analysed under light microscope at ×20 magnification. **b** Viability of RH30 and RD cells treated for 48 h with increasing concentration of Olaparib or AZD2461 expressed in relationship with mocked controls (DMSO), arbitrarily set at 100%, assessed by trypan blue exclusion staining. Each bar represents the mean value of three independent experiments ± SD. Statistical significance: *, *p* < 0.05, ***, *p* < 0.005, *ns* not significant vs. DMSO mocked controls. **c** Flow cytometry data showing percentages of cells in G1, S and G2 phases in RH30 and RD cells treated for 48 h with Olaparib (1.5 and 5 µM) or AZD2461 (5 and 10 µM). Data are average values of three independent experiments. Statistical significance was < 0.005 in both PARPi-treated RH30 and RD cells vs. mocked controls. **d** Western blot analyses of a panel of cell cycle regulatory proteins (Cyclin B1, Cyclin D1, p-Cdc2, Cdc25C and p21) in RH30 and RD cells at 48 h after exposure to PARPi. Tubulin expression was used as internal control. Representative blots of three independent experiments

The effects of Olaparib, able to block both PARP1 and PARP2 activity (Goulooze et al. 2016), and AZD2461, a newly synthesised PARP1/2/3 inhibitor (Boehler et al. 2011), were evaluated in RMS cell lines. Increasing concentration of Olaparib (1.5 and 5 µM) or AZD2461 (5 and 10 µM) clearly affected the morphologic appearance of both RH30 and RD cells, as confirmed by microscope acquisitions at 48 h, with cells becoming dose-dependently larger in comparison to the control cells (Fig. 2a). Furthermore, PARPi-treated cells showed a significant dose-dependent reduction in their number (Fig. 2b): direct counting for living cells using the trypan blue dye exclusion test showed that Olaparib exposure is able to inhibit cell growth by about 35% at 1.5 µM in both RMS cell lines, whilst higher concentrations (5 µM) reduced proliferation by 70% in RH30 cells and by 60% in RD compared to the untreated samples (Fig. 2b); AZD2461 led to about 35% decreased cell growth at 5 µM and to 60% at 10 µM in both RMS cell lines compared to mocked controls (Fig. 2b).

In order to determine whether the Olaparib- and AZD2461-dependent decreases in RMS cell growth were due to alterations in cell cycle progression, flow cytometry analysis was performed in RH30 and RD cells. Based on PI staining of cellular DNA content, cells significantly arrested in G2 phase (4n) when treated for 48 h with Olaparib or AZD2461 with a corresponding decrease of cell percentage in both G1 (2n) and S phases, whilst untreated cells rapidly divided and progressed through the cell cycle at high rates (Fig. 2c). Indeed, a maximum 4n-peak was observed at the higher drug concentrations (from 6.7 ± 1.7% in DMSO to 77.4 ± 2.8% in 5 µM Olaparib and 73.6 ± 2.5% in 10 µM AZD2461 RH30 cells; from 12.0 ± 2.7% in DMSO to 63.5 ± 2.4% in 5 µM Olaparib and 65.6 ± 2.1% in 10 µM AZD2461 RD cells), confirming a dose-dependent accumulation of cells in the G2/M phase in both RMS cell lines (Fig. 2c).

**Fig. 3:**
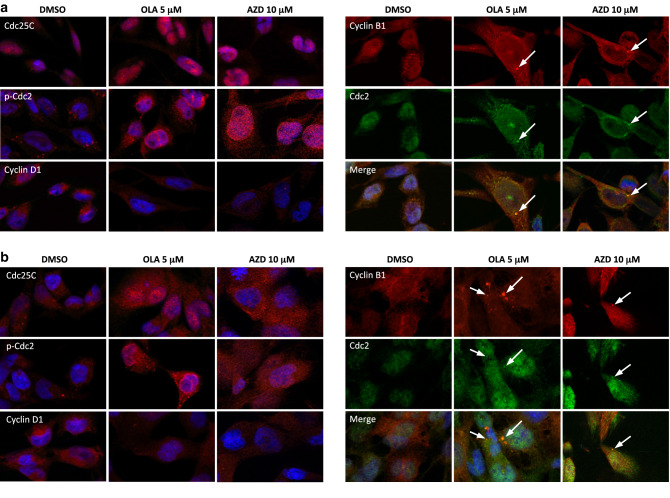
Changes in molecular regulators of cell cycle in RMS cells treated with Olaparib or AZD2461. **a** Immunofluorescence experiments showing the expression and localization of Cdc25C, p-Cdc2 (Thr14/Tyr15), Cyclin D1, Cyclin B1 and Cdc2 proteins in RH30 cells treated with 5 µM Olaparib or 10 µM AZD2461 for 48 h. Control cells were treated with DMSO. DAPI was used for nuclear staining. Images captured under ApoTome microscope at ×40 magnification. **b** Immunofluorescence experiments showing the expression and localization of Cdc25C, p-Cdc2, Cyclin D1, Cyclin B1 and Cdc2 proteins in RD cells treated with 5 µM Olaparib or 10 µM AZD2461 for 48 h. Control cells were treated with DMSO. DAPI was used for nuclear staining. Images captured under ApoTome microscope at ×40 magnification

To analyse the mechanisms underlying these cell cycle perturbations, the impact of Olaparib and AZD2461 on the expression and activation status of proteins related to cell cycle checkpoints was investigated. Western blotting experiments showed that the PARPi-mediated G2/M cell cycle arrest was associated with a dose-dependent upregulation of Cyclin B1, phospho (p)-Cdc2, Cdc25C and p21 proteins, as well as with a concomitant downregulation of Cyclin D1 levels in both RH30 and RD cells (Fig. 2d). IF experiments showed alterations in the expression, activation and sub-localization of cell cycle regulators in the RMS cell lines, especially at the maximum Olaparib (5 µM) or AZD2461 (10 µM) used dosages (Fig. 3a, b). Olaparib and AZD2461 single exposure for 48 h caused a cytoplasmatic retention of Cdc25C, a protein involved in the mitosis entry, in both RH30 and RD treated-cells (Fig. 3a, b). The marked up-regulation of Cdc2 phosphorylation levels at Thr14/Tyr15 in both cell lines treated with Olaparib or AZD2461 confirmed that the Cdc2 protein was in its inactive form (Fig. 3a, b). Cyclin D1 cytoplasmatic levels also resulted downregulated in PARPi-treated RH30 (Fig. 3a) and RD cells (Fig. 3b). Furthermore, Cdc2 and Cyclin B1, two G2/M-regulating proteins, co-localized outside the nuclear portion with a massive staining evident in the perinuclear area (Fig. 3a, b) in both PARPi-treated RMS cells. This finding suggests that the complex Cdc2/Cyclin B1 was no longer able to enter the nucleus, this leading to the G2/M arrest evidenced by FACS analysis.

Altogether, these observations demonstrate that Olaparib and AZD2461 display a rapid cytostatic effect in RMS cells, with an evident block of the cell cycle at G2/M phase.

### Prolonged treatment with Olaparib and AZD2461 induces apoptosis in RMS cell lines due to persistent DNA damage

**Fig. 4:**
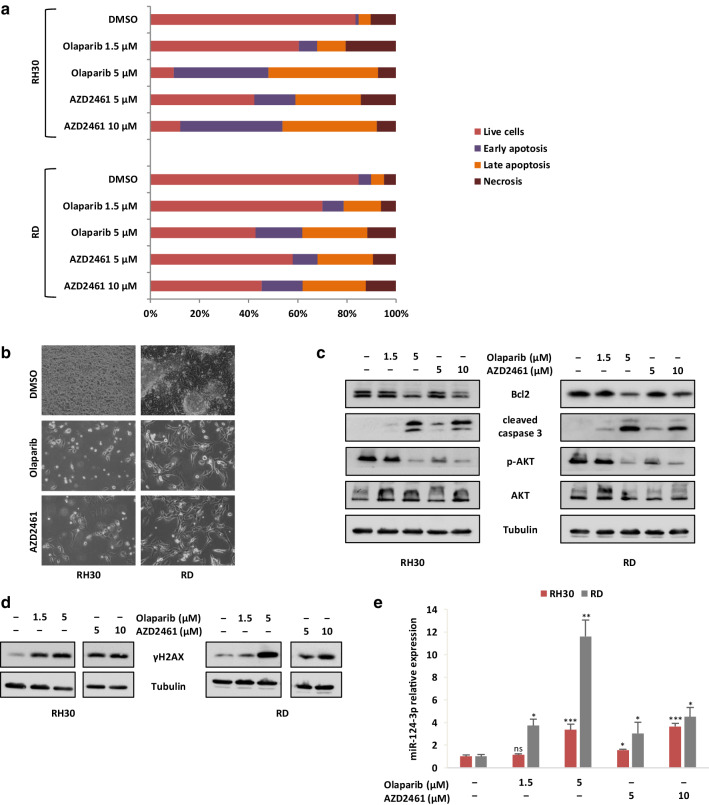
Effects of Olaparib and AZD2461 treatment on apoptosis in RMS cell lines. **a** Histograms show the rate of apoptosis in RMS cells 144 h after PAPRi treatment (1.5 and 5 µM Olaparib or 5 and 10 µM AZD2461). Cells were stained with Annexin V and 7-AAD and then analysed by flow cytometry. Data are expressed as a percentage of total cell number. **b** Images showing morphological changes observed in both RH30 and RD cells analysed under light microscope at 20x magnification at 144 h after 5 µM Olaparib or 10 µM AZD2461 treatment. Unlike mocked controls, RMS treated-cells exhibited a wide number of vacuoles and cytoplasm destruction. **c** Western blots showing the expression of the apoptosis related proteins, Bcl2, cleaved caspase-3, p-AKT (Ser473) and AKT in RH30 and RD cells after prolonged treatment with Olaparib or AZD2461. Tubulin expression was used as internal control. Representative blots of three independent experiments. **d** Expression levels of phospho-H2AX (γH2AX) analysed by western blotting experiments in RH30 and RD cells treated with Olaparib (1.5 and 5 µM) or AZD2461 (5 and 10 µM) for 144 h compared to mocked control cells (DMSO). Tubulin expression was used as loading control. Representative blots of two independent experiments. **e** q-PCR analysis of miR-124-3p mRNA levels in RH30 and RD cells treated with increasing concentration of Olaparib or AZD2461 for 144 h. The results are expressed as fold increase over relative mocked controls (DMSO), arbitrarily set at 1. Transcript levels were normalized to U6 mRNA and error bars represent SD of two independent q-PCR reactions, each performed in triplicate. Statistical significance: *, *p* < 0.05, **, *p* < 0.01, ***, *p* < 0.005, *ns* not significant vs. DMSO mocked controls

To assess whether the decreased cell growth of RMS cells treated with PARPi was also caused by the induction of programmed cell death, flow cytometry assays with Annexin V-PE/7-AAD staining were performed. Treatment of RH30 and RD cell lines with Olaparib (1.5 and 5 µM) or AZD2461 (5 and 10 µM) for 48 h did not increase the percentage of cells undergoing early or late apoptosis when compared to mock-treated control cells (data not shown). Olaparib or AZD2461 significantly increased the number of apoptotic RH30 and RD cells only after 144 h of treatment (Fig. 4a). Indeed, Annexin V-PE/7-AAD double staining confirmed that cytotoxic effects were most pronounced after more prolonged exposure times at higher drug concentration, with an increased percentage of apoptosis in both PARPi-treated RMS cell lines compared with controls (Fig. 4a). Cell death induced by long-lasting PARPi exposure was evident in the morphologic appearance of both RH30 and RD cells, which exhibited a wide number of vacuoles and cytoplasm destruction, whilst DMSO-treated RMS cells rapidly reached confluence and grew in multiple layers (Fig. 4b). Furthermore, Western blot analysis showed that Bcl2 levels clearly decreased in RH30 and RD cells at the higher drug concentrations (Fig. 4c), whilst caspase-3 cleavage/activation proportionally increased with PARPi concentrations and became strongly evident at 5 µM Olaparib and 10 µM AZD2461 in both RMS cell lines (Fig. 4c), in accordance with the apoptosis data obtained by FACS analysis. Since AKT molecular pathway is a pivotal signal in RMS apoptosis (Kilic-Eren et al. 2013), activation of AKT phosphorylation at Ser473 was analysed. As observed in Western blotting assays, phosphorylation levels of AKT protein (p-AKT) were markedly reduced in a dose-dependent way in both RH30 and RD cells treated with PARPi compared to the untreated controls, whilst AKT total levels were unchanged (Fig. 4c). Indeed, the treatment of RMS cells with LY29004, a synthetic molecule known to inhibit the PI3K/AKT axis, led to the downregulation of p-AKT expression and the concomitant upregulation of cleaved caspase 3 (data not shown), this confirming the central role of the AKT signal transduction pathway in the PARPi-mediated cell survival and death.

To assess if the activation of the cell death pathways was due to a permanent DNA damage, the accumulation of activated H2AX histone (γH2AX), a well-established marker for DSBs (Kuo and Yang 2008; Bonner et al. 2008), was evaluated in RH30 and RD cells treated with Olaparib (1.5 and 5 µM) or AZD2461 (5 and 10 µM). Western blotting assays demonstrated that the γH2AX levels remain significantly elevated in RH30 and RD treated-cells in comparison to mocked controls at 144 h (Fig. 4d), this suggesting that the persistence of DNA injury is the primary cause of the cellular lethality observed after prolonged exposure to PARPi. Since the downregulation of BCL2, the increased caspase 3 activation and the accumulation of γH2AX have been recently correlated to apoptosis by the upregulation of miR-124-3p (Zhang et al. 2017), we evaluated the expression of this miRNA in PARPi-treated cells. Indeed, miR-124-3p levels significantly and dose-dependently increased after Olaparib (1.5 and 5 µM) or AZD2461 (5 and 10 µM) exposure (Fig. 4e). These results suggest that the increased cell death triggered by PARPi, especially at higher doses, is related to different molecular components, comprehending miRNA modulation.

### Olaparib or AZD2461 in combination with IR reduces clonogenic capacity and increases DNA damage in RMS cell lines

**Fig. 5:**
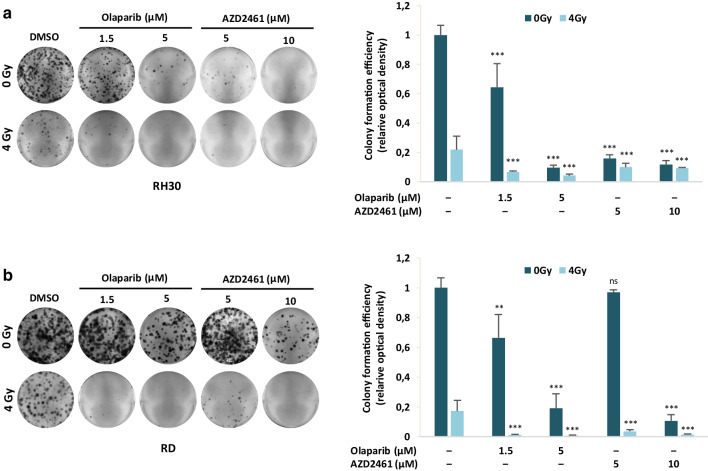
PARPi single exposure radiosensitizes RH30 and RD cells, and decreases clonogenic ability of RMS cells. RH30 and RD cells untreated (DMSO) or pretreated with Olaparib or AZD2461 for 24 h were irradiated or not with a single dose of 4 Gy. Four h after IR, cells were seeded at low concentration and allowed to grow for 12 days to examine their colony formation capacity. Representative pictures of RH30 (**a**) and RD (**b**) colonies stained with crystal violet. Colony forming efficiency was calculated by crystal violet absorbance from two independent experiments, each performed in triplicate. Each bar represents the means ± SD. Statistical significance: **, *p* < 0.01, ***, *p* < 0.005, *ns* not significant vs. DMSO mocked controls

**Fig. 6:**
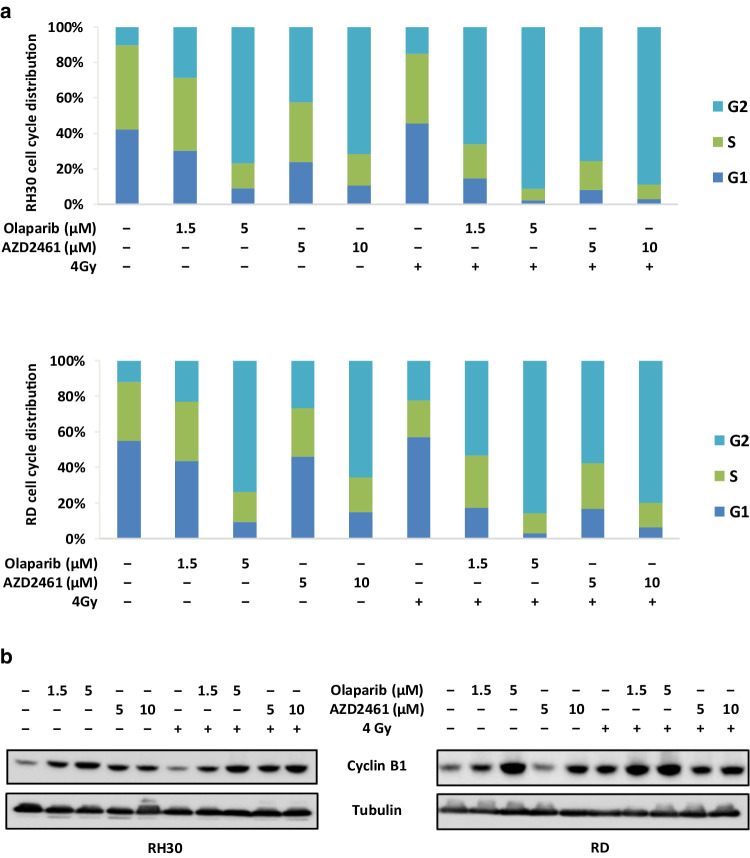
Olaparib and AZD2461 treatment in combination with IR induces a strong increase of RMS cells in G2/M phase. RH30 and RD cells untreated (DMSO) or pretreated with Olaparib (1.5 and 5 µM) or AZD2461 (5 and 10 µM) for 24 h were irradiated or not with a single dose of 4 Gy. Cells were incubated for additional 24 h at 37 °C. **a** Flow cytometry data showing percentages of RH30 and RD cells in G1, S and G2 phases. Data are average values of two independent experiments. **b** Western blot analyses of cell cycle regulatory protein Cyclin B1 in RH30 and RD cells 48 h after exposure to PARPi and 24 h after IR. Tubulin expression was used as the internal control. Representative blots of two independent experiments

**Fig. 7:**
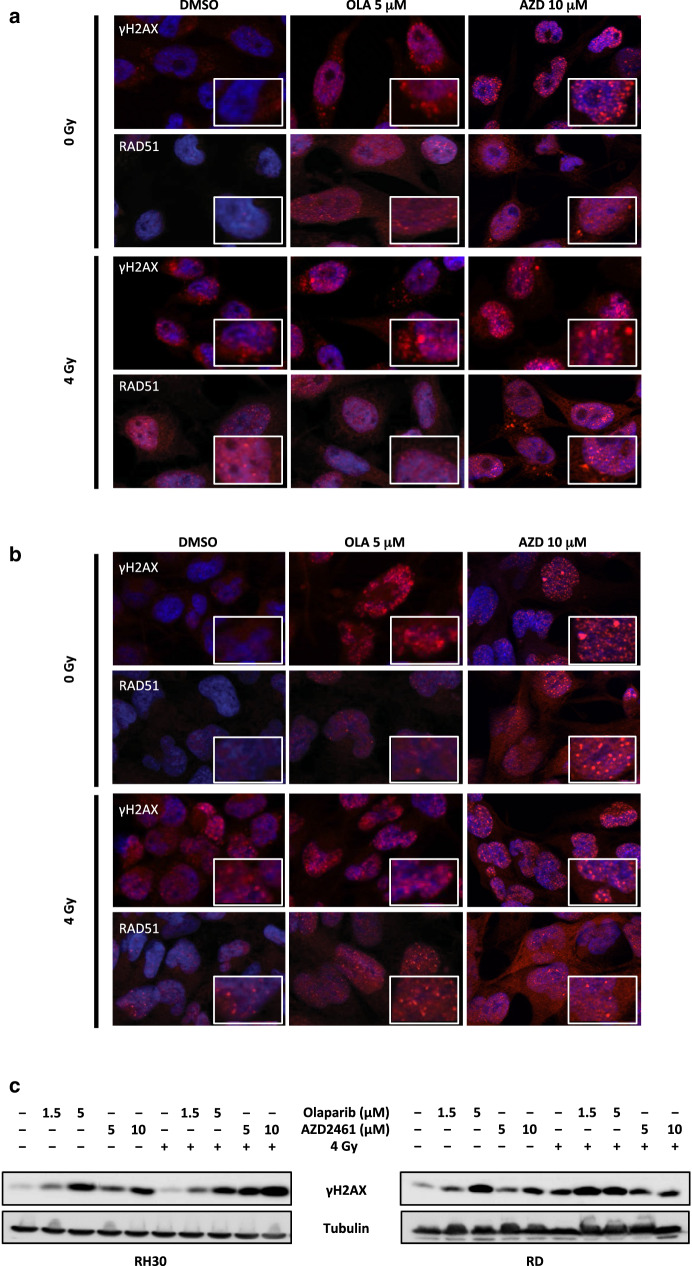
Exposure to Olaparib and AZD2461 increases DNA damage after radiation treatment in RMS cell lines. **a** RH30 cells, untreated (DMSO) or pretreated with 5 µM Olaparib or 10 µM AZD2461 for 24 h, were irradiated or not with a single dose of 4 Gy. Four h after IR, cells were fixed for immunofluorescence experiments. Expression and localization of γH2AX and RAD51 proteins were analysed. DAPI was used for nuclear staining. Images captured under ApoTome microscope at ×40 magnification. The square in each panel represents a magnification of γH2AX and RAD51 foci. **b** RD cells, untreated (DMSO) or pretreated with 5 µM Olaparib or 10 µM AZD2461 for 24 h, were irradiated or not with a single dose of 4 Gy. Expression and localization of γH2AX and RAD51 proteins were evaluated at 4 h after IR by immunofluorescence experiments. **c** RH30 and RD cells, pretreated or not with 1.5 and 5 µM Olaparib or 5 and 10 µM AZD2461, were lysed for total protein extraction at 24 h after 4 Gy irradiation. Western blots showing the expression levels of γH2AX protein. Tubulin expression was used as internal control. Representative blots of two independent experiments

Genotoxic damage, i.e., the formation of SSBs and DBSs in DNA molecules, is the most relevant mechanism by which IR causes cell-cycle arrest and cellular lethality (Vignard et al. 2013). To investigate the radiosensitising properties of Olaparib and AZD2461 in RH30 and RD cells, clonogenic survival assays were performed at 12 days after drug treatment with or without IR. The simultaneous treatment of either Olaparib (1.5 and 5 µM) or AZD2461 (5 and 10 µM) with IR (4 Gy) was more effective than the single exposure to each specific PARPi agent or to irradiation, this resulting in a significant reduction in colony formation capacity in both RH30 and RD cells (Fig. 5a, b), as assessed by crystal violet absorbance. Indeed, PARPi/IR combination led to a significant decrease (≥ 85%) in colony formation and the effect was more evident at the lower concentration of both Olaparib (1.5 µM) or AZD2461 (5 µM) (Fig. 5a, b). Cell cycle analysis, performed 24 h after IR in the presence or absence of Olaparib (1.5 and 5 µM) or AZD2461 (5 and 10 µM) pre-treatment, showed a significantly higher accumulation of cells in G2 phase after the combined exposure compared to only irradiated (4 Gy) cells or to cells treated with each drug concentration but not irradiated (Fig. 6a). In particular, the synergistic mechanism of action was clearly evident at the lowest doses of both PARPi agents: in RH30 cells (Fig. 6a upper graph), the combined exposure of 1.5 µM Olaparib/4 Gy led to a 2.3- and 4.4-fold increase of G2 cell percentage in comparison to 1.5 µM Olaparib (*p* = 0.013) and IR (*p* = 0.003) alone, respectively; a significant increase of G2 phase was observed in 5 µM AZD2461/4 Gy co-treatment in comparison to 5 µM AZD2461 (1.8-fold, *p* = 0.017) or radiation alone (5.0-fold, *p* = 0.003). In RD cells (Fig. 7a lower graph), 1.5 µM Olaparib/4 Gy combined administration resulted in a 2.3- and 2.4-fold increase of cell percentage at the G2 phase in comparison to 1.5 µM Olaparib (*p* = 0.023) and IR (*p* = 0.006) alone, respectively; a similar trend was obtained with 5 µM AZD2461/4 Gy compared to 5 µM AZD2461 (2.1-fold, *p* = 0.003) or IR alone (2.5-fold, *p* = 0.0023). Upregulation of Cyclin B1 also closely matched with the PARPi/IR-induced G2 cell cycle arrest in both RH30 and RD cell lines (Fig. 6b), this confirming that the cumulative effects were more notable by using 1.5 µM Olaparib or 5 µM AZD2461 in combination with 4 Gy exposure.

In order to assess whether Olaparib and AZD2461 exposure may sensitize RMS cells to IR by inducing DNA damage and impairing the DNA repair system, the abundance of γH2AX and RAD51, one important component of the HR machinery, was analysed. The IF experiments confirmed that PARPi treatment combined with IR for 4 h induces a greater number of γH2AX and RAD51 foci in both RH30 and RD nuclei than either PARPi or 4 Gy single exposure, especially at the maximum Olaparib (5 µM) or AZD2461 (10 µM) used dosages (Fig. 7a, b). Quantitation of γH2AX and RAD51 foci in response to the different drug and/or IR exposures is reported in Supplementary File 1. Western blots on RH30 and RD cell lysates, processed 24 h after a single dose of 4 Gy in the presence or in absence of Olaparib (1.5 and 5 µM) or AZD246 (5 and 10 µM), confirmed that DNA damage significantly persisted at later times post IR or PARPi treatments compared with control cells, with the most elevated levels of γH2AX being present in the cotreated samples (Fig. 7c).

Interestingly, a drastic alteration in the cell cycle distribution and anti-clonogenic effects were also observed when Olaparib or AZD2461 treatment was coupled with IR at 2 Gy, this suggesting that an effective anti-tumour activity in RMS cells is already achieved at a lower dosage of the different therapeutic modalities (Supplementary File 2).

Altogether, these results demonstrate that Olaparib and AZD2461 may sensitize RMS tumour cells to the irradiation.

## Discussion

RMS is the most frequent soft tissue sarcoma in childhood (McDowell 2003; O’Neill et al. 2013). Even if the survival probability has increased to about 70% for children and adolescents with RMS (Ognjanovic et al. 2009), the 5-year survival rate for patients with relapsed or metastatic disease is approximately 40%, mainly due to the development of chemo- and radioresistances (Wolden et al. 2015). Therefore, novel more effective therapeutic strategies are a pressing need, in those advanced patients.

In the present study, RH30 and RD cell lines—two in vitro models of ARMS and ERMS subtypes, respectively—were used to evaluate the cellular and molecular responses to the PARP inhibitors Olaparib and AZD2461, as single agents or in combination with IR. PARPi have strong cytotoxic effects in tumours harbouring genetic mutations in the components of the DDR system, such as BRCA1, BRCA2, PTEN and XCCR4, due to a mechanism indicated as “synthetic lethality”, according to which the inability to correct PARPi-induced SSBs leads to fatal DNA damage and cellular death (Brown et al. 2017). This study showed that the treatment with Olaparib, a specific inhibitor of PARP1 and PARP2 enzymes, or AZD2461, a newly PARP1/2/3 competitor, used as single agents, reduces cell proliferation in both RH30 and RD cells in a dose dependent manner, with a marked arrest in the G2/M phase of the cell cycle. In accordance with the flow-cytometry data, Olaparib or AZD2461-treated cells showed morphological alterations, such as an evident cell volume enlargement, which are characteristic of a defective cell division arrest (Fig. 2a). These observations are in agreement with data very recently reported by Mangoni et al. (2018). In the present study we examined in detail the molecular mechanisms of PARPi effects on cell cycle progression and survival. We showed that changes in cell cycle distribution are driven by the deregulation of specific regulatory markers: (1) PARPi treatment led to the downregulation of Cyclin D1 expression and to the overexpression of p21 cell cycle regulator; and (2), PARPi activated the G2/M checkpoint in RMS cells by sequestering Cdc25C in the cytoplasm compartment, promoting hyper-phosphorylation of Cdc2 (p-Cdc2) at Thr14/Tyr15, and upregulating Cyclin B1 levels. Specifically, Cyclin B1 protein resulted predominantly accumulated around the nuclear envelope, this confirming that high levels of p-Cdc2 are not able to form an active complex with Cyclin B1, a phenomenon that prevents their entrance in the nucleus and stalls the mitosis process as reported in other cancer types (Jin et al. 1996). Concerning cell survival, PARPi prolonged exposure led to apoptosis through the inhibition of AKT activation and the modulation of relative downstream molecules. Indeed, reduced levels of phospho-AKT and Bcl2 proteins, with the concomitant cleavage and activation of the caspase-3 protein were observed in PARPi-treated cells. The present findings also demonstrate that the cell survival impairment we observed is linked to the accumulation of the DNA damage, which PARPi-treated cells are unable to repair. Phosphorylation of histone H2AX on serine 139 to form γH2AX, a sensitive marker for the indirect quantification of DNA DSBs (Bonner et al. 2008), was not only evident after 48 h of drug exposure but was significantly increased after 144 h in both Olaparib and AZD2461-treated cells, this explaining the observed severe consequences on the RMS cell survival. Indeed, persistence of γH2AX signal (Löbrich et al. 2010) correlated with an inefficient reconstitution of the DNA integrity, which is essentially performed by the HR signalling molecules in the G2/M phase (Polo and Jackson 2011). Finally, the increased expression of miR-124-3p in Olaparib- or AZD2461-treated RMS cells suggests that the modulation of this miRNA is involved in the complex molecular mechanisms underlying the PARPi-mediated cytotoxicity. Interestingly, low expression of miR-124-3p has been observed in different types of human cancers, including RMS (deep sequencing data reported in Megiorni et al. 2014), and the restoration of miR-124-3p levels is able to decrease cell survival by promoting apoptosis (Wang et al. 2016; Ma et al. 2018; Zhang et al. 2018). The molecular mechanisms and pathways related to the functions of miR-124-3p in RMS models will be further investigated.

Effects on cell proliferation, apoptosis and DNA damage were more pronounced by using higher concentrations of both drugs, i.e., 5 µM Olaparib and 10 µM AZD2461, and mainly in RH30 cells. Since BRCA1/BRCA2 mutations have not been found in RMS tumours (Mendes-Pereira et al. 2009), the presence of genetic/epigenetic alterations in other DNA repair machinery components or related factors cannot be excluded. To this regard, the expression of PTEN gene, which encodes for a protein involved in DNA DSB repair, is commonly suppressed in both ARMS and ERMS tumours (Zhu et al. 2016). The importance of PTEN deficiency in the synthetic lethality driven by PARP inhibitors has been described in several cancers (Mendes-Pereira et al. 2009; Dedes et al. 2010; McEllin et al. 2010; He et al. 2015). Therefore, the higher sensitivity to Olaparib or AZD2461 observed in RH30 cells compared to RD cells might partially be explained by the more pronounced down-regulation of PTEN levels in the alveolar compared to the embryonal cell line (our preliminary data not shown), this underlying the usefulness of PARPi treatment in tumours with deregulated HR-linked proteins, such as PTEN, independently to the BRCA1/2 status alone. A different possible mechanism underlying the more conspicuous PARPi-mediated effects in ARMS cells might be related to the high levels of MYCN protein detectable in RH30 but not in RD cells (our preliminary data not shown). These findings are consistent with previous studies in neuroblastoma (NB) tumours, showing that PARPi lead to DNA damage and cell death more effectively in MYCN-amplified than in MYCN-not-amplified NB cell lines (Bridges et al. 2014; Verhagen et al. 2015). MYCN gene has been demonstrated to sustain DNA damage by delaying the resolution of DNA lesions (Venere et al. 2014), which if not properly repaired can lead to cellular death, this establishing a mechanistic link between MYCN overexpression and sensitivity to PARP inhibition.

Notably, the present study supports the possibility to combine PARPi with standard treatments, in particular with radiotherapy, in patients with RMS. Since ionising radiations induce DNA breaks that require PARP activity for proper DSB repair, PARP inhibition provides an effective tool to make cancer cells more radiosensitive. Previously, in vitro and in vivo studies have reported that a series of PARPi is able to radiosensitise different tumour models, including breast cancer, glioblastoma, neuroblastoma and lung cancer (Mueller et al. 2013; Bridges et al. 2014; Venere et al. 2014; Verhagen et al. 2015). In the present study, the synergistic antitumour activity of the combined treatment of PARPi and IR was demonstrated by the evidence that the exposure to Olaparib or AZD2461 radiosensitises RMS cells by amplifying the quantity and the duration of DNA damage induced by IR. The susceptibility of tumour cells to ionising radiations was synergistically enhanced through the increased accumulation of chromatid-type breaks, the prolonged activation of the G2/M checkpoint and the reduction of clonogenic potential in both RH30 and RD cell lines. Cell cycle regulation has been reported as an important biological mechanism affecting radiosensitivity, with cells being most sensitive to radiation during the G2/M phase (Pawlik and Keyomarsi 2004). Likewise, different drugs have been shown to promote sensitivity to radiations by inducing cells to accumulate in this phase (Pawlik and Keyomarsi 2004; Duangmano et al. 2012). Our in vitro experiments demonstrated that Olaparib or AZD2461 plus radiations significantly increase γH2AX and RAD51 expression and nuclear accumulation, this further confirming that the combined treatment has a higher sensitizing activity compared to either treatment modality. The increase of γH2AX and RAD51 foci is suggestive of the presence of stalled fork recovery sites and endogenous replication stress, both signs of the attempt by tumour cells to repair DNA lesions (Costanzo 2011; He et al. 2015). RAD51 upregulation contributes to G2 phase arrest in order to help the HR systems in fixing DNA damage, but an excess of RAD51 nuclear foci is thought to promote genomic instability for inappropriate recombination events, including translocations and other rearrangements, this in turn having deleterious effects on cell survival. Indeed, the sustained overexpression of RAD51 nuclear signals has been associated with a reduced cell growth and apoptosis, as observed in Drosophila as well as in human cell lines (Flygare et al. 2001; Yoo and McKee 2004; Klein 2008), which confirms that a balanced interaction between RAD51 and other HR factors is needed to properly repair DNA.

A further interesting finding that emerges from our data is that low concentrations of either PARPi (1.5 µM Olaparib or 5 µM AZD2461) are adequate to increase substantially the efficacy of the ionising radiations in both RH30 and RD cell lines, this having potentially important clinical implications, since a significant level of tumour cell radiotoxicity can be achieved by more tolerable concentrations of PARPi and IR in combination. However, further studies will be needed to evaluate the antitumour activity and possible toxicity of Olaparib and AZD2461 as single agents and in combination with ionising radiations in RMS xenografts.

In conclusion, the present findings demonstrate that PARPi may represent a promising therapeutic approach in RMS treatment. Furthermore, PARPi-increased sensitivity to radiations may be associated with a significant therapeutic benefit by inhibiting tumour growth and survival and by counteracting the development of radioresistance, this potentially improving clinical outcome.

## Electronic supplementary material

Below is the link to the electronic supplementary material.

Supplementary File 1 PARPi treatment in combination with IR exposure increases γH2AX and RAD51 foci in RMS cells. RH30 and RD cells untreated (DMSO) or pretreated with Olaparib (5 μM) or AZD2461 (10 μM) for 24 h were irradiated or not with a single dose of 4 Gy. Four h after IR, cells were fixed for immunofluorescence experiments. Histograms show fluorescence intensity of phospho-H2AX (γH2AX) and RAD51 in RH30 (a) and RD (b) cell lines in the respect of cell number in each analysed field. The results are expressed as fold increase over mocked control cells (DMSO) arbitrarily set at 1 (PDF 63 KB)

Supplementary File 2 Synergistic effects of PARPi and 2 Gy exposure on RMS growth and clonogenicity. RH30 and RD cells untreated (DMSO) or pretreated with Olaparib (1.5 and 5 μM) or AZD2461 (5 and 10 μM) for 24 h were irradiated (IR) or not with a single dose of 2 Gy. After IR, cells were incubated for additional 24 h at 37°C for cell cycle analysis and 4 h at 37°C for clonogenic assay (a) Flow cytometry data showing percentages of RH30 and RD cells in G1, S and G2 phases. Data are average values of two independent experiments. (b) Cells were seeded at low concentration and allowed to grow for 12 days to examine their colony formation capacity. Representative pictures of colonies stained with crystal violet (PDF 167 KB)
