# Supplementary figures and images for: PARP inhibitors affect growth, survival and radiation susceptibility of human alveolar and embryonal rhabdomyosarcoma cell lines
Source: J Cancer Res Clin Oncol. 2018 Oct 24;145(1):137–52. doi: 10.1007/s00432-018-2774-6 (PMC6326011; doi:10.1007/s00432-018-2774-6)

**a** **$\gamma$ H2AX**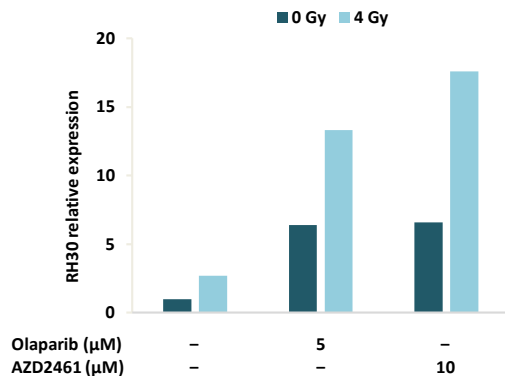**RAD51**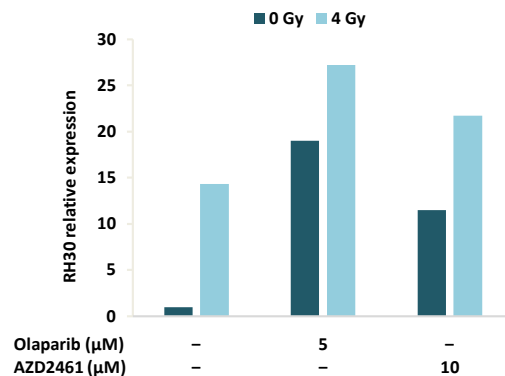**b** **$\gamma$ H2AX**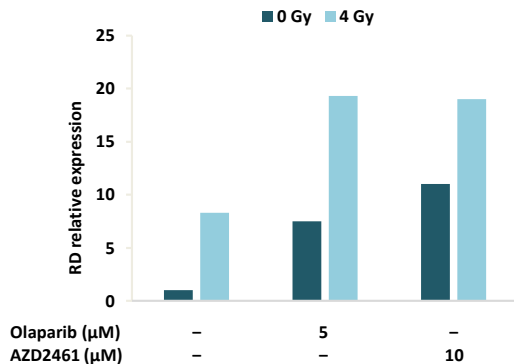**RAD51**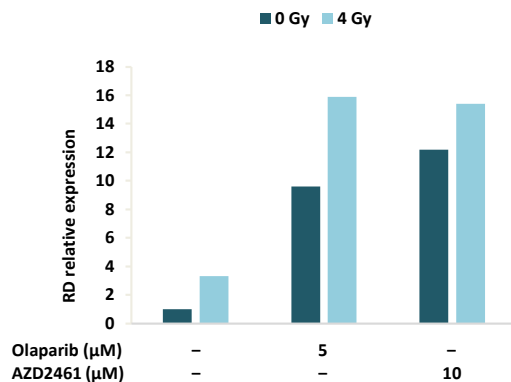

Supplement: Supplementary file 1 — PARPi treatment in combination with IR exposure increases γH2AX and RAD51 foci in RMS cells. RH30 and RD cells untreated (DMSO) or pretreated with Olaparib (5 μM) or AZD2461 (10 μM) for 24 h were irradiated or not with a single dose of 4 Gy. Four h after IR, cells were fixed for immunofluorescence experiments. Histograms show fluorescence intensity of phospho-H2AX (γH2AX) and RAD51 in RH30 (a) and RD (b) cell lines in the respect of cell number in each analysed field. The results are expressed as fold increase over mocked control cells (DMSO) arbitrarily set at 1 (PDF 63 KB) [file 432_2018_2774_MOESM1_ESM.pdf]
