# Supplementary material for: PARP inhibitors affect growth, survival and radiation susceptibility of human alveolar and embryonal rhabdomyosarcoma cell lines
Source: J Cancer Res Clin Oncol. 2018 Oct 24;145(1):137–52. doi: 10.1007/s00432-018-2774-6 (PMC6326011; doi:10.1007/s00432-018-2774-6)
Supplement: Supplementary file 2 — Synergistic effects of PARPi and 2 Gy exposure on RMS growth and clonogenicity. RH30 and RD cells untreated (DMSO) or pretreated with Olaparib (1.5 and 5 μM) or AZD2461 (5 and 10 μM) for 24 h were irradiated (IR) or not with a single dose of 2 Gy. After IR, cells were incubated for additional 24 h at 37°C for cell cycle analysis and 4 h at 37°C for clonogenic assay (a) Flow cytometry data showing percentages of RH30 and RD cells in G1, S and G2 phases. Data are average values of two independent experiments. (b) Cells were seeded at low concentration and allowed to grow for 12 days to examine their colony formation capacity. Representative pictures of colonies stained with crystal violet (PDF 167 KB) [file 432_2018_2774_MOESM2_ESM.pdf]

a

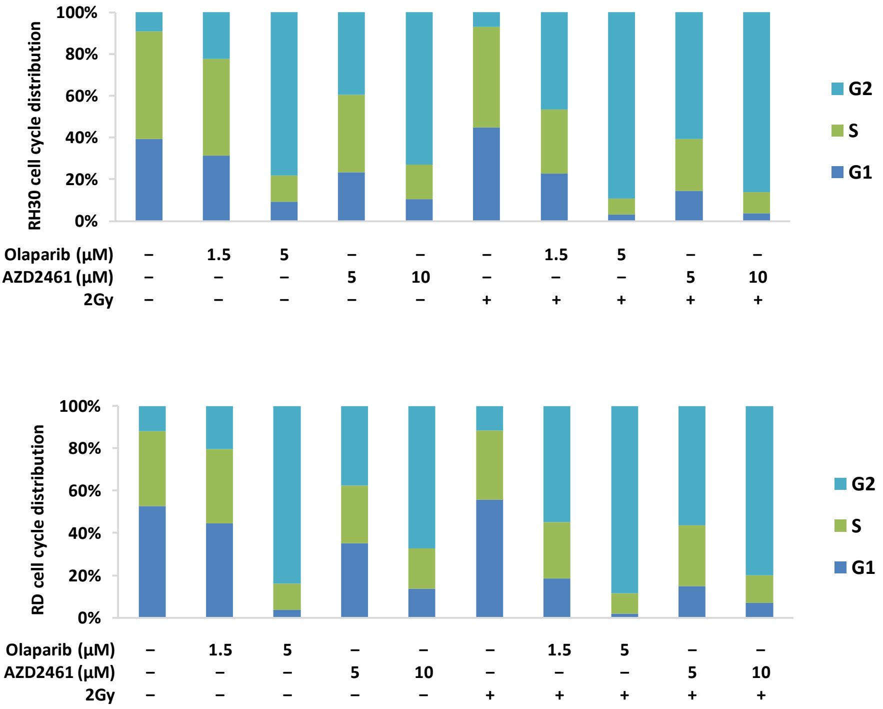
RH30 cell cycle distribution
100%
80%
60%
40%
20%
0%
G2
S
G1
Olaparib (μM) – 1.5 5 – – – 1.5 5 – –
AZD2461 (μM) – – – 5 10 – – – 5 10
2Gy – – – – – + + + + +
RD cell cycle distribution
100%
80%
60%
40%
20%
0%
G2
S
G1
Olaparib (μM) – 1.5 5 – – – 1.5 5 – –
AZD2461 (μM) – – – 5 10 – – – 5 10
2Gy – – – – – + + + + +

b

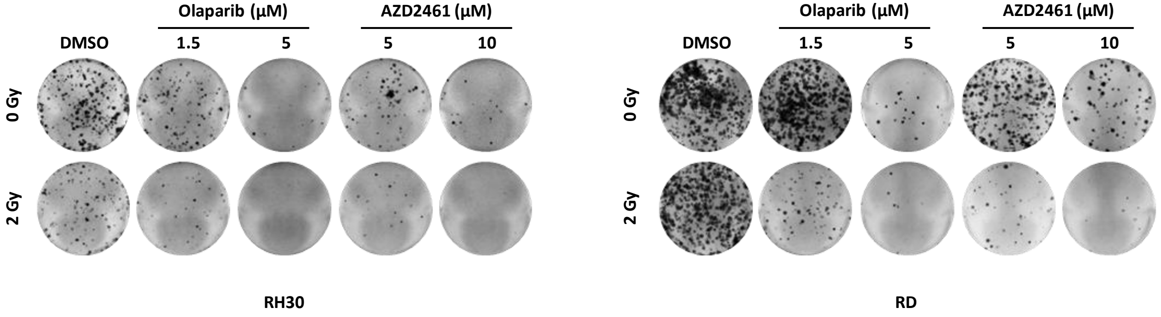
Olaparib (μM)
AZD2461 (μM)
DMSO
1.5
5
5
10
0 Gy
2 Gy
RH30
Olaparib (μM)
AZD2461 (μM)
DMSO
1.5
5
5
10
0 Gy
2 Gy
RD

Article title: PARP inhibitors affect growth, survival and radiation susceptibility of human alveolar and embryonal rhabdomyosarcoma cell lines.

Journal name: Journal of Cancer Research and Clinical Oncology.

Author names: Simona Camero, Simona Ceccarelli, Francesca De Felice, Francesco Marampon, Olga Mannarino, Lucrezia Camicia, Enrica Vescarelli, Paola Pontecorvi, Barry Pizer, Rajeev Shukla, Amalia Schiavetti, Maria Giovanna Mollace, Antonio Pizzuti, Vincenzo Tombolini, Cinzia Marchese, Francesca Megiorni and Carlo Dominici. Simona Camero, Simona Ceccarelli, Francesca De Felice, Francesco Marampon, Olga Mannarino, Enrica Vescarelli, Paola Pontecorvi, Barry Pizer, Rajeev Shukla, Amalia Schiavetti, Maria Giovanna Mollace, Antonio Pizzuti, Vincenzo Tombolini, Cinzia Marchese, Francesca Megiorni and Carlo Dominici.

Corresponding author: Francesca Megiorni, PhD - Department of Paediatrics, "Sapienza" University of Rome.
Viale Regina Elena 324
00161 Rome, Italy. E-mail: francesca.megiorni@uniroma1.it
